# The ReFiBot makers guide: Fostering academic open science and circularity with a robotic educational kit

**DOI:** 10.1016/j.ohx.2023.e00484

**Published:** 2023-11-04

**Authors:** Christos Pantos, Jurrian Doornbos, Gonzalo Mier, João Valente

**Affiliations:** a Information Technology, Wageningen University & Research, Wageningen, The Netherlands; b Laboratory of Geo-information and Remote Sensing, Wageningen University & Research, Wageningen, The Netherlands

**Keywords:** Robotics, Education, Circularity, Open-science, Recycling, Low-cost

## Abstract

The advent of robotics in schools and universities curricula are preparing students to encompass new didactic fields. This article presents ReFiBot which is an education robot that has been used to increase the technical literacy on robotics and bring more awareness to open science at Wageningen University. The ReFiBot combines open-source hardware and software, integrated with a chassis made from recycled plastic from fishnets. The ReFiBot was carefully designed to be easily assembled with off-the-shelf electronic parts and programmed using the Arduino IDE. Moreover, a software library is facilitated to ease its adoption in educational activities from any curricula level. The ReFiBot has been mainly used for education but can also be used for research on swarm robotics. The CAD files, components list, software files, and tutorial within this contribution will guide the reader through the assemblage and best practices of this circular robotics kit.

## Graphical abstract

**Figure:**
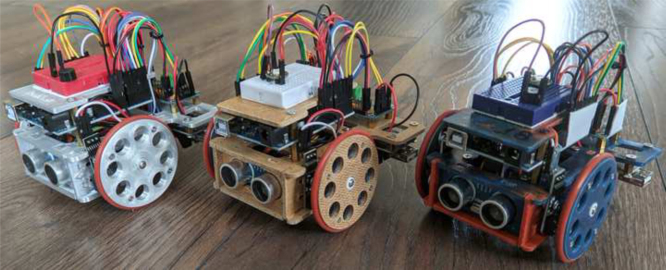

**Table:** 

Hardware name	ReFiBot
Subject area	• Educational Tools and Open Source Alternatives to Existing Infrastructure
Hardware type	• Robotics
Open source license	GNU General Public License v3.0
Cost of hardware	35 €
Source file repository	https://doi.org/10.5281/zenodo.7823461
	or https://github.com/saidlab-team/RefiBot

## 1 Hardware in context

The demand for more Computer Science knowledge across all domains ought to the clear, in light of lagging or missing legislation on the subject, increased reliance on information technologies, and high job demand in the field. One method of engaging potential and existing Computer Science students is by having a more hands-on experience. Examples exist where the introduction of an educational robot into the coursework improved engagement [1], increased motivation [2], and directed future intentions for the students [2]. These improvements have been measured to different extents in students across all levels of education [3]: from elementary [4] and high-school [5], to undergraduate [6], [7] and graduate students [8], [9]. More engaged and motivated students in Computer Science have direct and indirect benefits of helping the students better understand the subject matter curing coursework, or possibly steering them towards STEM-related interests for future educational and vocational decisions [5]. Educational robotics kits serve to increase the hands-on experience of students and could be aimed at many different levels. Such as the LEGO Mindstorms [7] for elementary and high school, the turtlebot family for teaching the Robotics Operating System [10] or the kits from Pérula-Martínez et al. [6] and López-Rodríguez and Cuesta [11]. However, these kits are not without limitations, such as closed ecosystems in hardware and software [9], [6], expensive hardware [6], [10], [12] or difficult reconfigurability [9], or developed with a different mechanical principle in mind [13], such as a robotic arm. Table 1 shows a comparison between similar wheeled robots, aimed at education or makers as found in literature. From the available options, not a single system, however, fulfills the following requirements: open-sourced software and hardware, a wide range of sensors, manufacturable from recycled materials, and can be assembled by non-experts following provided instructions. This paper presents ReFiBot (Recycled Fishnet Bot), an educational robotics kit, which focuses on Open Source Hardware and Software, whilst improving sensing capabilities and manufacturability at a low cost. ReFiBot can be made from recycled materials (in our case recycled fishnet plastic), whilst maintaining modularity to adapt other sensors and features to fit in any learning environment.

**Table 1:** Comparison between open-source educational robots.

Platform	Cost	Description	Features	Educational value
Robotont [14]	2000 €	Omni-directional, autonomous, wheeled robot	RGB-D camera, onboard computation, ROS	Technical education, computer vision, ROS
TurtleBot 3 [10]	500 €	Skid-steering, autonomous wheeled robot	RGB-D camera, onboard computation, ROS	Technical education, computer vision, ROS
Andruino A1 [11]	35 € (w/o smartphone)	Skid-steering, smartphone-controlled wheeled robot	Internet connectivity, low-cost	Technical education, networking, Arduino, basic programming
Omni [12]	550 €	Omni-directional, autonomous, wheeled robot	RGB-D camera, onboard computation, obstacle avoidance, shock absorption	Technical education, computer vision, ROS, mechanical engineering
**ReFiBot**	**35 €**	**Skid-steering Arduino wheeled robot**	**Recycled materials, low-cost, simple to make many ReFiBots**	**Technical education, Arduino, basic programming, sensor interfacing.**

**Fig. 1:**
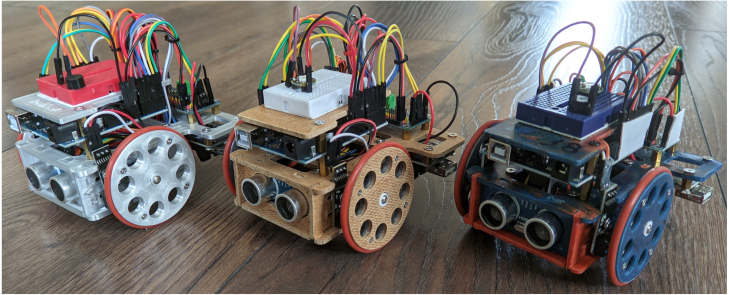
Fully assembled ReFiBot with a chassis made from three different materials (from left to right): aluminum, wood, and recycled plastic.

## 2 Hardware description

The ReFiBot (Recycled Fishnet Robot) is an educational three-wheel, differential drive robot (Fig. 1). The design has been developed for teaching robotics principles: from the assembly of a small robot to the process required to program it. Moreover, ReFiBot is made with inexpensive components and is distributed with detailed instructions on the building process, which encourages professors and students to build their own ReFiBot. The most distinguishing features are as follows:

• Easily manufactured and assembled using a variety of methods, catering to each maker’s preferred technique (3D-printing, CNC-machining, laser-cutting, etc.)
• A range of sensors and actuators to learn robotics principles and demonstrate the possibilities of low-cost robots.
• Open source and beginner-friendly programming environment using the Arduino [15] platform.
• Affordable and easily obtainable components.

**Fig. 2:**
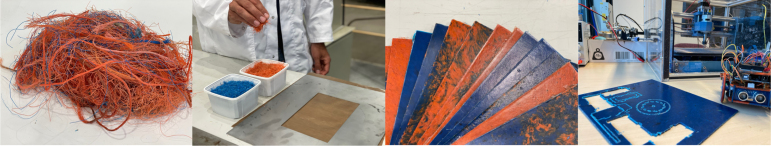
The complete chain from fishing net to a new recycled product. From the left to the right: The fishing net, placing it shredded into the sheet mold, the end product, and the some individual parts cut.

Due to the flat designs of all the parts, it can be manufactured using many different modalities: such as 3D printing, by CNC machining of wood, plastic sheets or aluminum plates, or even laser cutting. For our ReFiBots, plastic sheets made from fishnets were used (Fig. 2). The fishnets were made from Nylon. The fishnets were shredded and melted into a liquid state. Afterward, they were flattened by a metal mold to form small sheets of 200 mm × 300 mm × 3 mm. Using a small CNC router, these sheets were cut into parts for ReFiBot. We recommend consulting an expert in polymers for the proper handling of melted plastic. Some polymers including nylon release hazardous fumes. This is why ReFiBot can also be made from any sheet material, an example of aluminum and wood is shown in Fig. 1. Additionally, 3D printing ReFiBot with PLA plastic is also a less hazardous alternative.

**Fig. 3:**
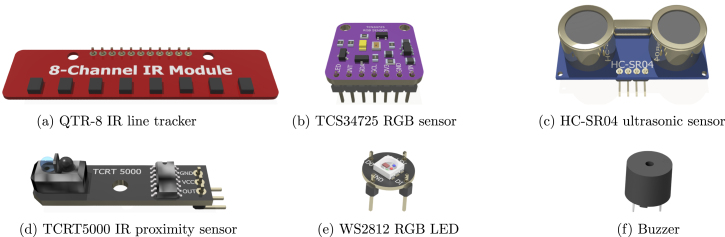
Sensor boards overview.

All electronics are off-theshelf parts to keep the cost as low as possible while they can be sourced from most electronics hobby stores. The robot is equipped with various sensors and actuators, as Fig. 3 illustrates.

Fig. 3(a): QTR-8 IR line tracker — When an object is placed in front of the sensor, the infrared light from the emitters is reflected back to the receptors and the light intensity is measured. The readings are affected by the color, the distance, and the surface of the object. Typically, this particular sensor is used in robotics to lines below the robot.

Fig. 3(b): TCS34725 RGB sensor — The TCS34725 board is a tiny, one-pixel camera that is capable to sense and quantify the color values of the point in front of the sensor. This sensor is also equipped with a white LED to ensure sufficient light intensity for taking good measurements.

Fig. 3(c): HC-SR04 ultrasonic sensor — This sensor measures the distance to the closest object, by measuring the time needed for the sound wave emitted to be received. Using a transmitter, which sends an ultrasonic pulse burst (outside of our hearing spectrum), and a receiver, waiting for this signal to hit the sensor.

Fig. 3(d): TCRT5000 IR proximity sensor — This infrared sensor is used to measure the velocity of the wheel. By emitting one of the IR sensors and calculating the intensity of the reflection in the other IR sensor. A low reflection is an opening in the wheel, and a high reflection is a closed area in the wheel. By tracking the time between these events, the program calculates the velocity of the robot.

Fig. 3(e): WS2812 RGB LED — The robot is also equipped with an RGB LED to produce visual feedback effects in specific situations. This module has three embedded LEDs and each one emits in a different part of the light spectrum (red, green, blue). The color and the brightness can be controlled individually using only one pin while multiple LEDs can be connected in a chain configuration.

Fig. 3(f): Buzzer — Similarly to the LED, the users can use the buzzer to produce sound feedback signals. A typical application, for instance, is when the robot is approaching an obstacle where different sounds can indicate how close the robot is to this object.

**Fig. 4:**
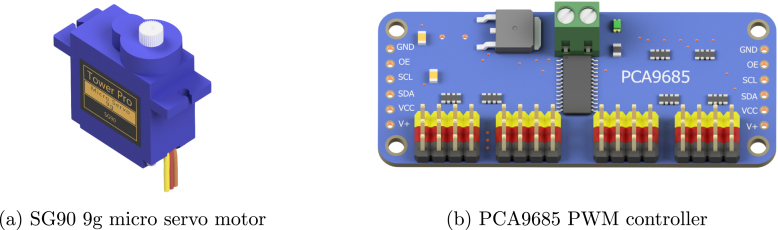
Robot wheel driving modules.

The robot uses two micro servo motors (Fig. 4(a)) in a continuous rotation configuration (360° rotation) to power the wheels. The stall torque of the servos at 4.8 V is 0.1765 Nm while their nominal operating speed is 0.12 s/60°. Considering the wheel diameter is around 4.8 cm and the total weight is roughly 300 grams, the theoretical acceleration is 2.75 ${\mathrm{m/s}}^{2}$. The maximum rotation speed of the SG90 is 120 RPM, resulting in a theoretical max speed of around 0.3 m/s For driving the servo motors a PCA9685 module (Fig. 4(b)) is used which is capable to control up to 16, 12-bit, PWM (Pulse Width Modulation) channels individually requiring only two pins. Although the robot requires only two channels for the servos, the users can use the spare channels to drive other modules that require PWM signals such as LEDs.

**Fig. 5:**
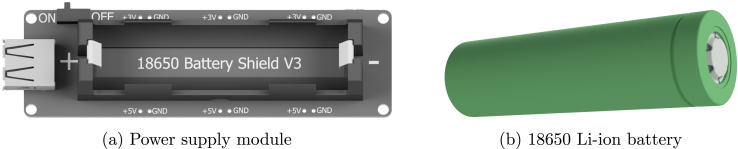
Robot power supply.

The system is powered by an off-the-shelf power supply module using a 18650 Li–ion Battery (Fig. 5). This module has an over-charge or over-discharge protection to charge safely the battery. However, there is no reverse polarity protection, and the users must make sure that the battery is correctly installed. Otherwise, the module will be damaged. On its output, a set of pin headers provide 3 and 5 V and the nominal current output is 1 and 3 Amperes respectively. In addition, a type A female USB connector that users can use to power other peripherals if necessary. Please refer to Section 5.12 for more details on proper battery handling. Additionally, alternatives such as Alkaline and LiFePo4 were considered but were deemed unsuitable for ReFiBot due to re-usability, cost, availability, or size constraints. A recommendation for future iterations is to implement a reverse polarity circuit into the battery supply module.

## 3 Design files

The robot chassis was designed to enable the manufacturing of the main pieces out of flat sheets with a thickness of 3 mm. The same design files can be used with almost any material type based on the user’s preference and manufacturing capabilities. However, it is recommended to use non-conductive materials such as plastic or wood to eliminate the risk of accidentally causing a short circuit that can damage the electronics. In Table 2 the design files, the .step files, and the .stl files are presented. The *Ball Holder* is the only part that cannot be made of flat sheets, therefore access to a 3D printer is required.

**Table 2:** Design files to build the chassis of the ReFiBot.

Design filename	File type	Link	Preview
Arduino protector	.ipt, .stl, .stp	Arduino Protector	
Ball holder	.ipt, .stl, .stp	Ball Holder	
Bottom plate	.ipt, .stl, .stp	Bottom Plate	
Front plate	.ipt, .stl, .stp	Front Plate	
Left Wheel	.ipt, .stl, .stp	Left Wheel	
Right wheel	.ipt, .stl, .stp	Right Wheel	
M3 Spacer	.ipt, .stl, .stp	M3 Spacer	
Side plate	.ipt, .stl, .stp	Side Plate	
Top plate	.ipt, .stl, .stp	Top Plate	

## 4 Bill of materials

The bill of materials is organized into three sections for improved readability. Table 3 compiles the essential electronic components required for constructing the circuit. Lastly, Table 4 contains the mechanical elements, such as screws or wires. Should be noted that for the *Left Wheel*, *Right Wheel*, and *Side plate* two units are needed to build the chassis.

Finally, all the components shown in Table 2 can also be manufactured using a 3D printer. The area necessary for producing the robot chassis with fishnet recycled plastic is 0.04 $m^{2}$, and the associated cost depends on the price of the electricity and labor time needed to melt the plastic waste. Conversely, if the parts are 3D printed using PLA (22 €/300 m) at a 20% infill and low quality, 16 m of filament would be required, taking 6.8 h and costing 1.30 €. However, employing a 100% infill and high quality would necessitate 26.3 m of filament, with a duration of 10.8 h and a cost of 1.90 €.

**Table 3:** Bill of electronic materials.

Designator	Component	Quantity	Cost per unit (€)	Total cost (€)	Supplier	Material
Arduino Uno	Arduino UNO R3 (USB cable included)	1	6.82 €	6.82 €	AliExpress	Composite
Breadboard	Mini Breadboard	1	0.62 €	0.62 €	AliExpress	Composite
QTR-8	8-Channel IR Line Tracker	1	2.07 €	2.07 €	AliExpress	Composite
PCA9685	PCA9685 16-Channel Servo Driver	1	3.20 €	3.20 €	AliExpress	Composite
TCS34725	TCS34725 RGB Sensor	1	1.61 €	1.61 €	AliExpress	Composite
HC-SR04	HC-SR04 Ultrasonic Proximity Sensor	1	0.77 €	0.77 €	AliExpress	Composite
SG90	SG90 Micro Servo (360°)	2	2.06 €	4.12 €	AliExpress	Composite
Buzzer	5 V Buzzer	1	0.20 €	0.20 €	AliExpress	Composite
WS2812	WS2812 (Neopixel) RGB LED	1	0.71 €	0.71 €	AliExpress	Composite
TCRT5000	TCRT5000 IR Proximity Sensor	2	0.46 €	0.92 €	AliExpress	Composite
Power Supply	18650 Battery Power Supply	1	1.77 €	1.77 €	AliExpress	Composite
Battery	Panasonic NCR18650 3400 mAh Li–ion Battery	1	2.75 €	2.75 €	AliExpress	Composite
Resistor	100 Ω Resistor	1	0.02 €	0.02 €	AliExpress	Metal
Jumper Wire	Female–Female 10 cm	3	0.05 €	0.15 €	AliExpress	Composite
Jumper Wire	Male–Male 10 cm	3	0.06 €	0.18 €	AliExpress	Composite
Jumper Wire	Female–Male 10 cm	6	0.06 €	0.36 €	AliExpress	Composite
Jumper Wire	Female–Female 20 cm	6	0.06 €	0.36 €	AliExpress	Composite
Jumper Wire	Female–Male 20 cm	16	0.07 €	1.12 €	AliExpress	Composite

**Table 4:** Bill of mechanic materials.

Designator	Component	Quantity	Cost per unit (€)	Total cost (€)	Supplier	Material
Flat head screw	Flat head screw M2 × 6 mm	10	0.02 €	0.20 €	AliExpress	Steel
Flat head screw^a^	Flat head screw M2 × 8 mm	2	–	–	AliExpress	Steel
Flat head screw	Flat head screw M3 × 6 mm	30	0.02 €	0.60 €	AliExpress	Steel
Flat head screw	Flat head screw M3 × 10 mm	2	0.04 €	0.08 €	AliExpress	Steel
Flat head screw	Flat head screw M3 × 12 mm	3	0.05 €	0.15 €	AliExpress	Steel
Hex nut	Hex nut M3	1	0.02 €	0.02 €	AliExpress	Steel
Hex locknut	Hex locknut M3	3	0.11 €	0.33 €	AliExpress	Steel, Nylon
Heat-set insets	Heat-set insets M2 × 3.2 × 3 mm	8	0.13 €	1.04 €	AliExpress	Brass
Hex threaded standoff	Hex threaded standoff M3 × 6 mm (FF)	8	0.03 €	0.24 €	AliExpress	Brass
Hex threaded standoff	Hex threaded standoff M3 × 12 mm (FF)	2	0.04 €	0.08 €	AliExpress	Brass
Hex threaded standoff	Hex threaded standoff M3 × 25 mm (FF)	4	0.31 €	1.24 €	AliExpress	Brass
Hex threaded standoff	Hex threaded standoff M3 × 12 mm (FM)	2	0.08 €	0.16 €	AliExpress	Brass
O-Ring	O-Ring 48 × 40 × 4mm	2	0.35 €	0.70 €	AliExpress	Silicone
Ball	Ball 12 mm	1	0.37 €	0.37 €	AliExpress	Steel
Servo horn^a^	Micro Servo Horn	2	–	–	AliExpress	Polymer

a Usually, it is part of the servo motor.

Table 3 on electronics indicates a total cost of 25.95 €, whereas the mechanical parts (Table 4) have a sum of 4.79 €. Resulting in 30.74 € for off-the-shelf parts. Depending on which materials, and price fluctuations, a single ReFiBot costs around 35 euro. Not including shipping costs of parts.

## 5 Build instructions

### 5.1 Conventions and tips

Before diving into the building instructions, some tips and conventions are provided in order to improve the user experience during the assembly process:

• The sides of the parts that have the word “ReFiBot” should always be face up.
• Before starting the assembly process prepare all the components and make sure there are no missing or broken parts. Also, check that all electronic modules have attached header pins.
• Follow the assembly steps in the same order as presented in this guide to avoid confusion and potential mistakes.
• Illustrations are enumerated in the order that each part should be assembled. Although there are some exceptions, this is the main rule.
• Screws and other threaded parts should not be tightened very hard. Specific torque values are not provided since they depend on the properties of the chosen materials.
• In every step, you will find illustrations that show the required parts, and instructions on how and where the components are mounted. Finally, a reference of the final result will be provided.
• By design, the same chassis parts have holes in various shapes where they can be used for installing the cables and accessing some components in case of troubleshooting.
• The characteristics and specifications of the modules presented in this work may differ from the modules you have. Please refer to the official data sheets of the devices you use.
• For safety reasons, never connect the battery or any other power source during the assembly. Please refer to Section 5.12 for additional information about safety.
• The robot chassis is divided into five main sub-assemblies. Every sub-assembly consists of several components, therefore verify that the correct parts are used as shown in the instructions in the illustrations.

### 5.2 Required tools

**Fig. 6:**
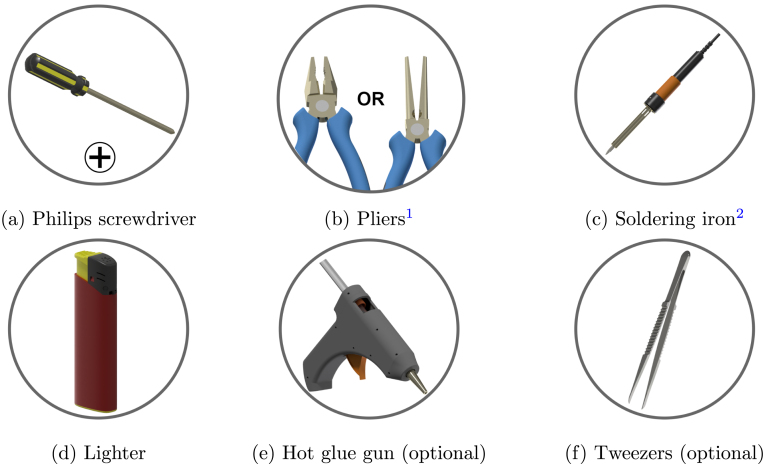
Tools required to assemble the robot. 1: Long-nose pliers is preferred; 2: Temperature-controlled soldering iron is preferred.

Tools that are required to assemble successfully the robot are illustrated in Fig. 6:

### 5.3 Wiring and electronics

Before starting the assembly of the robot body, the electronics should be prepared. Using the soldering iron, solder the required header pins on all electronic components. Soldering the jumper wires directly to the modules is not recommended, as it will hinder the replacement of a component if needed. At this point, jumper wires should be attached to the QTR-8, the TCS34725, and the HC-SR04 modules.

In general, the jumper wires are available in three different configurations: Female–Female (FF), Male–Male (MM) and Female–Male (FM). During this work, only two wire length were employed: 10 and 20 cm. To simplify the wiring process a wire color code is defined. For instance, the red wire indicates positive 5 V, black wire is the ground, yellow wire is the SDA pin, green wire is the SCL pin and the rest of the colors are assigned to the remaining pins. Table 5 summarizes the color code and the length required to wire all the electronics.

**Fig. 7:**
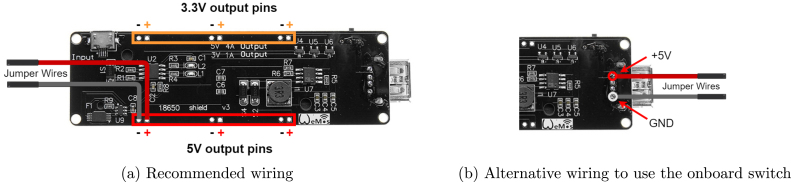
Battery power supply wiring configurations.

Once the jumper wires are attached to the modules QTR-8, TCS34725 and HC-SR04, attach the jumper wires to one of the three 5 V pin pairs of the battery power supply (Fig. 7(a)). Similarly to the previous step, the battery power supply module should be equipped with header pins before connecting the jumper wires. Usually, this module is equipped with a main power switch that activates/deactivates all available outputs. However, there are some versions of these modules where the switch controls only the output of the USB (type A) port. If this option is preferred, the wires should be soldered directly on the output of the USB port^1^ (Fig. 7(b)).

**Fig. 8:**
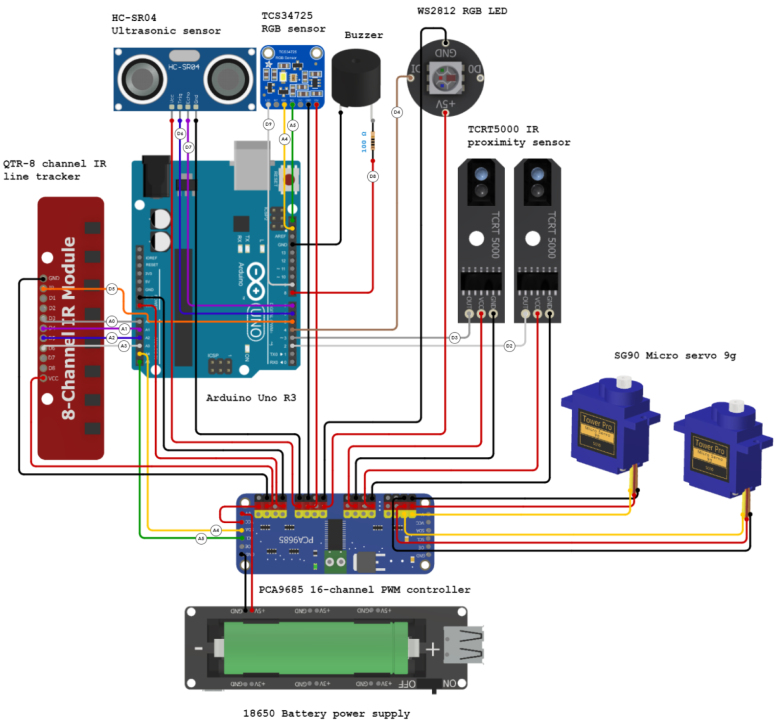
Complete wiring diagram of the robot peripherals.

The rest of the components should be connected in later stages of the assembly. The complete wiring diagram can be seen in Fig. 8. To secure the wires and prevent them from getting loose over time, you can fix them with hot glue.

**Table 5:**
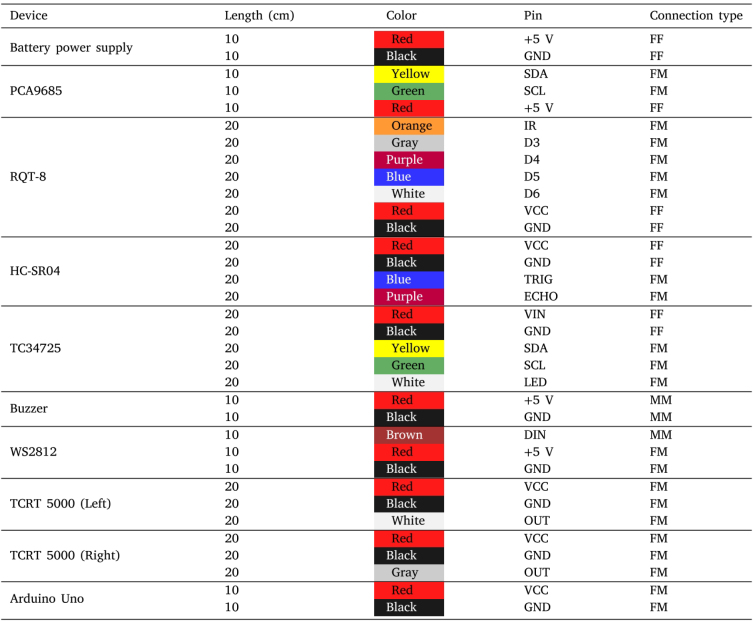
Wire color code.

### 5.4 Top chassis plate

**Fig. 9:**
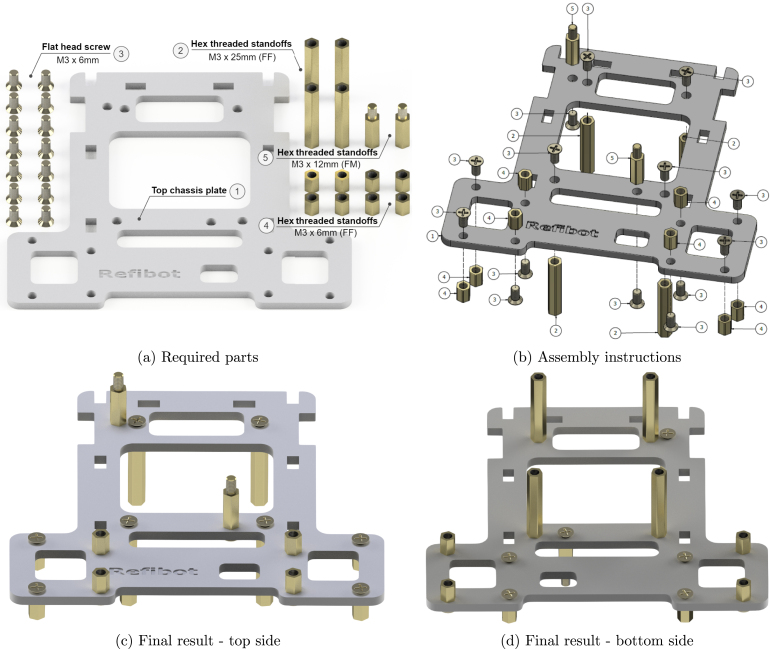
Top chassis plate sub-assembly.

The steps to assemble the top plate are illustrated in Fig. 9. The screws and standoffs required are shown in Fig. 9(a). Use a Phillips screwdriver (Fig. 6(a)) to mount the spacers to the top plate using M3 screws (Fig. 9(b)). The top chassis after the assembly process is illustrated in Figs. 9(c) & 9(d).

### 5.5 Bottom chassis plate

**Fig. 10:**
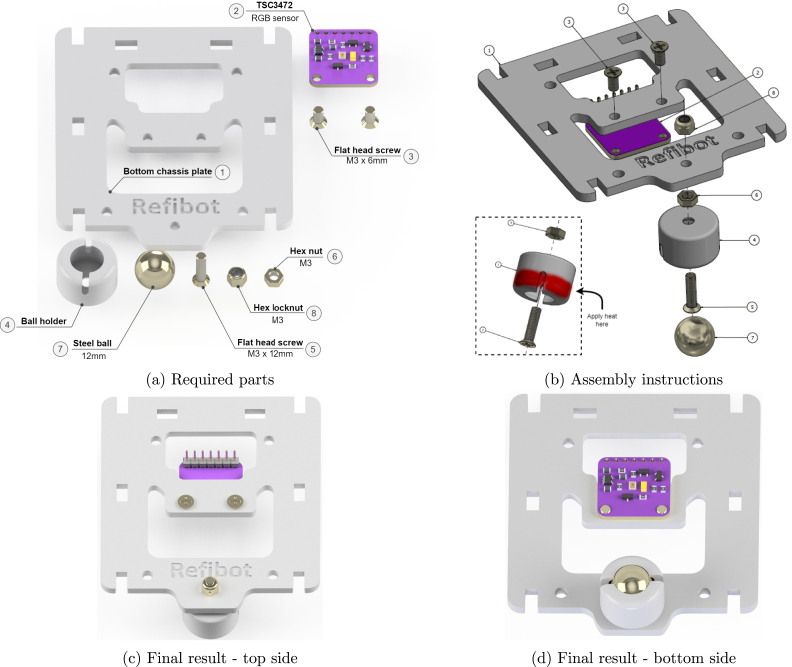
Bottom chassis plate sub-assembly.

To assemble the bottom part of the chassis (Fig. 10(a)), use the bottom plate and attach the TSC3472 module (Fig. 3(b)) with the help of the two M3 × 6 mm flat head screws. For simplicity, the cables are not illustrated in the figures and it is assumed that the jumper wires have been attached as described in Section 5.3. The next step is to assemble the omni-wheel. Pass an M3 × 12 mm flat head screw through the hole of the ball holder and secure the screw with an M3 hex nut. Tighten the screw firmly. In order to insert the steel ball in the ball holder, apply some heat on the outer shell of the holder as shown in Fig. 10(b), and then push the steel ball into the cavity. Assuming PLA is used as printing material, only 3–4 s are needed to soften the plastic with a common lighter (Fig. 6(d)). In case another material type is used, apply heat accordingly. To finish the bottom chassis plate assembly, attach the omni-wheel to the bottom chassis plate using an M3 lock nut. Use the illustrations in Fig. 10, Fig. 10 as a reference for the final result.

### 5.6 Side chassis plates

**Fig. 11:**
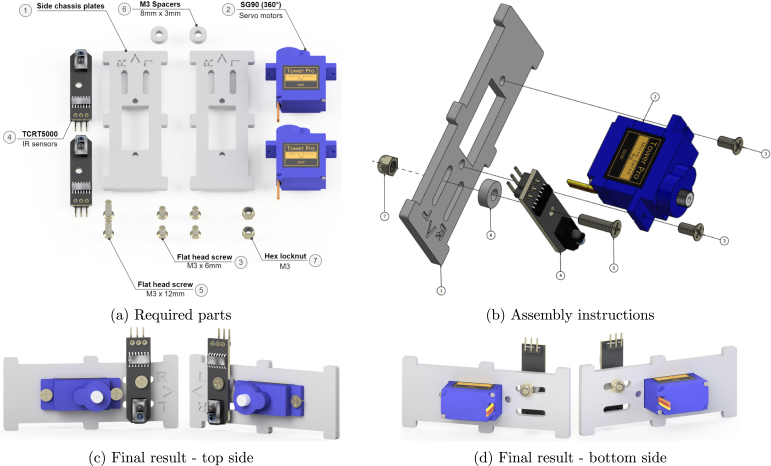
Side chassis plates sub-assembly.

Next, assemble the two side plates where the servos and the TCRT5000 IR sensors (Fig. 3(d)) are attached (Fig. 11(a)). Insert the servo motors in the designated slots as Fig. 11(b) indicates and secure them with M3 × 6 mm flat head screws. Please note, that the orientation of the servos also matters, the motor axle should be placed towards the front arrow. The next step is to mount the TCRT5000 sensors using an M3 × 12 mm flathead screw, an M3 spacer, and an M3 lock nut. The letters “L” (left) and “R” (right) on the chassis plate indicate the slot where the IR odometry sensor should be attached depending if it is on the left or the right side of the robot. This results in two side chassis plate assemblies such as in Fig. 11(c) for the front side and Fig. 11(d) for the backside. There are some useful tips below:

• The side on which there are engraved symbols must face outwards. Also, the plates should be installed in a specific orientation. The arrow should point at the front of the robot where the ultrasonic sensor is mounted.
• If the servos do not fit properly, avoid trying to force them due to the risk of damaging the cables. Instead, use a file to remove some material and safely install them.
• If soft material is used for the robot chassis, such as wood or soft plastic, it might be difficult to tap tighten the screws properly. In this case, longer screws should be used, such as M3 × 10 mm,^2^ in combination with M3 hex nuts to secure the servos.

### 5.7 Front chassis plate

**Fig. 12:**
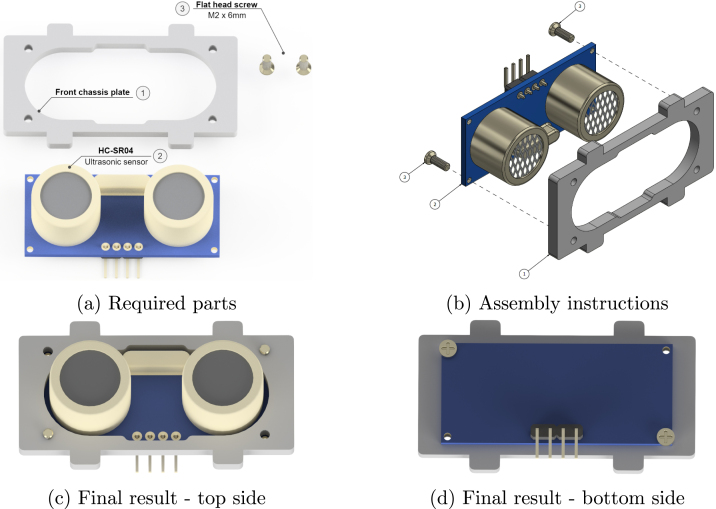
Front chassis plate sub-assembly.

The next sub-assembly is the front plate where the ultrasonic sensor is attached (Fig. 12(a)). Two screws will suffice for this assembly and are screwed into opposite corners (Fig. 12(b)). This results in the front and back of this front assembly as shown in Figs. 12(c) & 12(d).

### 5.8 Wheels

**Fig. 13:**
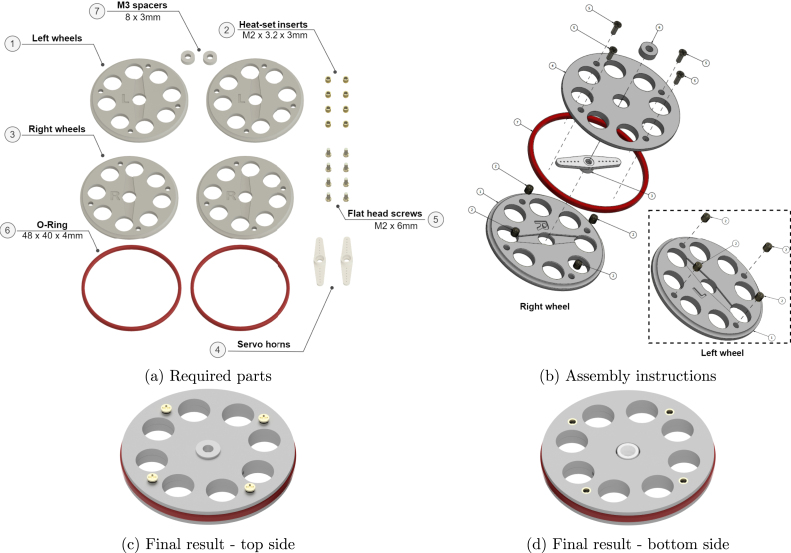
Wheels sub-assembly.

The last sub-assembly is the wheels. Each wheel is made of two disks: the left and the right, with the “R” and “L” letters engraved on the top surface respectively (Fig. 13(a)).

To assemble the two robot wheels successfully, the right and left, require slightly different layering. For the right wheel (Fig. 13(b) on the left), install the heat-set inserts on the four holes of the “R” disk. Assuming the disks are made of a polymer with thermoplastic properties, placed the disk on a flat surface and with a soldering iron set to the right temperature, press the inserts gently in the holes [16], [17]. The exact temperature depends on the material properties but as an indication for PLA, which is one of the most common 3D printed materials, 150–170 °C are enough to soften the plastic and secure the inserts. If another material type is used, such as metal, threads can be created directly in the holes and the inserts can be omitted. Alternatively, M2 hex nuts can be used instead, but longer screws must also be used. These modifications may require some additional components or changes to the CAD files.

In order to place the servo motor horn in the cavity of the same disk, verifying the flat side of the horn is facing upwards to the “L” disk. If the horn is too long and it blocks the wheel holes, it can be trimmed with pliers (Fig. 6(b)) or scissors. The holes must be wide open to avoid miss-readings by the TCRT5000 module (Fig. 3(d)).

The third step is to place the “L” on top of the “R” disk and secure it by using four M2 × 6 mm flat head screws. Finally, place the M3 spacer in the cavity of the wheel axis and then place the rubber gasket on the groove around the wheel. To assemble the left wheel the same steps should be followed, but the heat-set inserts should be installed on the “L” disk instead, as shown in Fig. 13(b) (right). Ensure that you have two mirroring versions of the wheels, where the screw-heads are on opposite ends, a single wheel final example is presented in Figs. 13(c) & 13(d).

### 5.9 Main body assembly

**Fig. 14:**
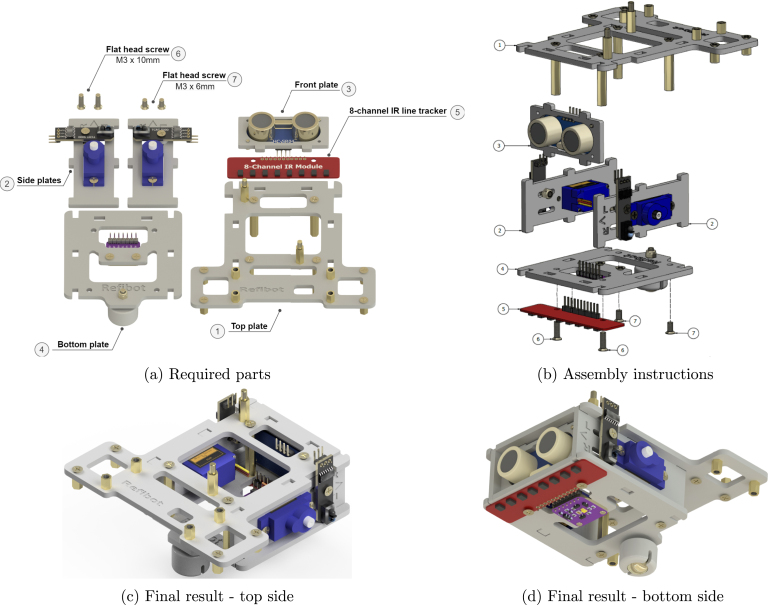
Main chassis assembly.

Using the sub-assemblies built on the previous steps (Fig. 14(a)), the main body of the robot can be assembled following the instructions in Fig. 14(b). The holes of the top plate can be used to access the components during the assembly and to organize the wires of the electronics. This results in the chassis assembly as shown in Figs. 14(c) & 14(d).

### 5.10 Electronics body assembly

**Fig. 15:**
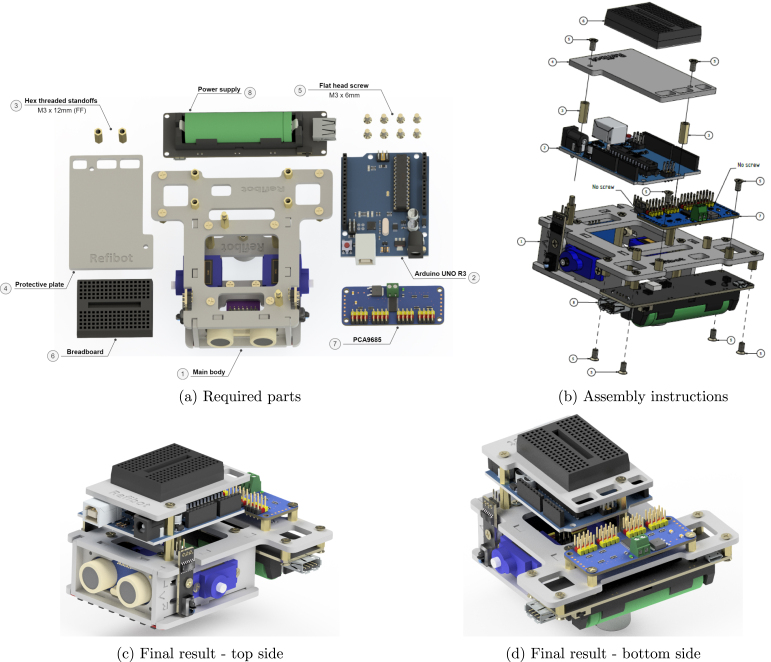
Electronics assembly.

At this stage, the rest of the electronics should be attached to the main body assembly (Fig. 15(a)). The Arduino Uno, PCA9865, power supply, and breadboard are screwed on their respective standoffs (Fig. 15(b)). This results in the final result assembly, as shown in Figs. 15(c) & 15(d). Verify that the battery or any other power source is not connected to any of the electronics before attaching them to the main body assembly.

The remaining wires should be connected following the schematic in Fig. 8. For better cable management, the power wires of the sensors are connected to the GND and +5V pins of the unused PWM channels of the PCA9685 module (Fig. 4(b)). The holes on the protective plate can be used also for easier wire management. To secure the breadboard on the protective plate, use the double side adhesive tape which is already attached to the bottom side of the breadboard. To prevent the wires of getting loose over time you can fix them with hot glue.

### 5.11 Attach the wheels

**Fig. 16:**
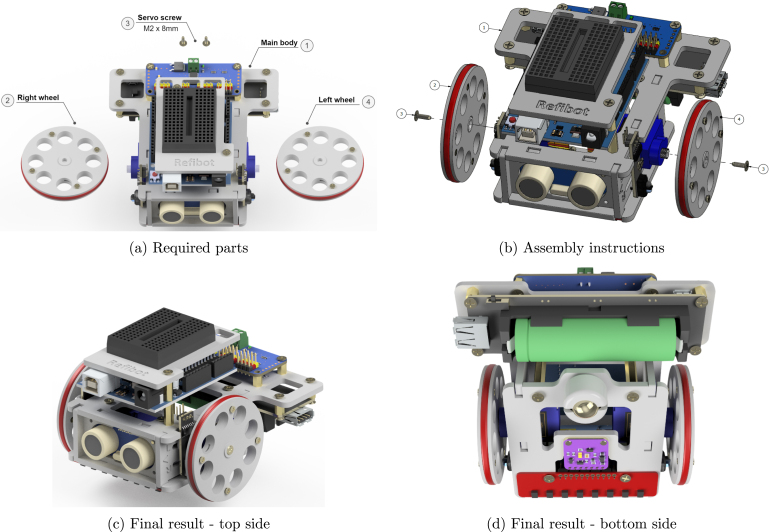
Final assembly.

After wiring up the ReFiBot, attach the wheel to the final assembly using the M2 × 8 mmm screws (Figs. 16(a) & 16(b)). The robot is completely assembled and should look similar to Figs. 16(c) & 16(d). Ready for testing and programming.

### 5.12 Battery charging and safety hazards

In general, Li–ion batteries can be dangerous if they are not handled properly. Therefore, before proceeding further, please consider the following potential safety hazards while using and charging the battery.

**Safety hazard disclaimer regarding Li–ion batteries:**

• Never short-circuit a Li–ion battery due to explosion risk.
• Do not expose the battery to heat or fire.
• Do not solder wires directly on the battery poles using a soldering iron. This can degrade the life span of the battery, and, in some cases, it can cause short-circuit.
• Do not drop, scratch, or remove the protective battery wrap.
• Do not leave the battery unattended while charging.
• Never use a battery if there are visible flaws such as deformations or the battery is swollen. In such cases, remove the battery from the power supply module carefully and dispose of it in one of your local battery recycling points.

The maximum power consumption of the system is around 400 mA while all peripherals are in use continuously. Since the robot is powered by a 3.7 V, 18650 Li–ion Battery (Fig. 5(b)) with a nominal capacity of 3400 mAh, the theoretical run time is around 8.5 h. The theoretical minimum is 8.5 h, and verified during assembly on a single ReFiBot. However, other peripheral use, battery quality, and battery condition all affect the potential duration of ReFiBot. This is why we present 4 h to be a minimum. In the classroom tutorials, we had all the ReFiBots last for the full tutorial duration of 4 h. This value is therefore the minimum verified duration for all the ReFiBots we have used.

When the robot assembly and the wiring are finished, the battery can be placed in the power supply module. Insert first the negative pole in the battery holder, and then the positive one. Be aware that the power supply module used in this work does not have protection against reverse polarity. Double-check that the battery is installed correctly. Otherwise, the module will be damaged. In case you want to remove the battery from the battery holder, remove first the negative pole and then the positive one.

To charge the battery, a common smartphone charger can be used by connecting a USB cable to the micro USB port of the power supply module. The USB of the power supply module is used only for charging the battery. For programming the robot, the Arduino’s USB port should be used instead. The red LED indicates that the battery is charging. When the battery is fully charged, the LED turns blue. The charging time is around 2–3 h but it depends on the output of the phone charger.

## 6 Operation instructions

Before beginning to program the ReFiBot, it is necessary to ensure that the battery is correctly positioned in the battery holder as explained in Section 5.12, and the Arduino USB cable connects the ReFiBot to the computer.

Once the ReFiBot is activated and connected to the computer, proceed with the installation of the libraries *Adafruit Bus IO* [18], *Adafruit NeoPixel* [19], *Adafruit PWM Servo Driver Library* [20], *Adafruit TCS34725* [21], and *QTRSensors* [22] into the Arduino [15] programming environment. Additionally, the integration of the library *“Awesome Lib”*, as supplied in GitHub repository https://github.com/saidlab-team/RefiBot or Zenodo https://doi.org/10.5281/zenodo.7823461 is required. This library has been designed to facilitate straightforward access to the sensors and actuators of the ReFiBot system. To check if the software installation has been done properly, the following code example is provided:

**Figure:**
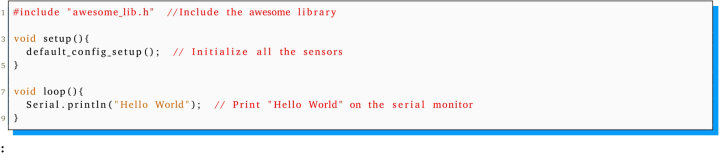

The capabilities provided by the *“Awesome Lib”* can be divided into six categories (Table 6). The first category relates to time management, facilitating the ability to wait for a specified duration or a signal received via the serial monitor. The remaining five categories are delineated based on the sensor or actuator impacted, enabling the operation of motors, the acquisition of data from the TCS34725 RGB color sensor, the extraction of information from the HC-SR04 ultrasonic sensor, the generation of sounds with the buzzer, or the measurement of the position of the black line above the ReFiBot using the QTR-8 IR line tracker.

Subsequent to the successful compilation and programming of the ReFiBot in the Arduino IDE, it may be detached from the Arduino USB cable, thereby enabling the autonomous operation of the robotic system. It is noteworthy that the disconnection of the USB cable results in the cessation of serial communication. Therefore, remove the cable exclusively when the employment of the serial monitor is not relevant on the ongoing tasks.

**Table 6:** List of functions provided on the *“Awesome Lib”* to control the ReFiBot.

Sensors	Functions	Explanation
Time management	void wait(float n_secs)	Wait ***n_secs*** seconds
	void wait_for_serial_input()	Wait until the serial monitor receives something
QTR-8 IR line tracker (Fig. 3(a))	uint16_t read_line_black_position()	Return the position of the black line under the RefiBot
	bool* black_line_array()	Read the sensor values
	int array_count(bool* arr)	Return the number of sensors that see black under the robot
TCS34725 RGB sensor (Fig. 3(b))	float* read_rgb_sensor()	Return three values with the color of the point in front of the camera, coded as [R,G,B].
HC-SR04 Ultrasonic sensor (Fig. 3(c))	float get_sonar_distance()	Return the distance (cm) from the sensor to the object in front of it.
Buzzer (Fig. 3(f))	void buzzer_sound(float time)	Make a 100 Hz sound for ***time*** seconds
	void buzzer_number(int n_beeps)	Beep ***n_beeps*** times
Motors (Fig. 4(a))	void stop_robot()	Stop both motors
	void go_front()	Move both motors at same speed
	void go_back()	Move both motors backward at same speed
	void go_right()	Move left motor forward faster than right motor
	void go_left()	Move right motor forward faster than left motor

The code block below shows an example of a simple behavior written with the library. It counts the number of walls/obstacles it has detected. Starting with the detection of something in front of the sonar sensor at a distance larger than 20 cm, the robot moves forward until the next sensor reading. But if the distance is equal to or less than 20 cm, it stops, beeps (for the number of wall counts), moves backward, and rotates right. After this, it loops back to the decision process to re-evaluate the distance.

**Figure:**
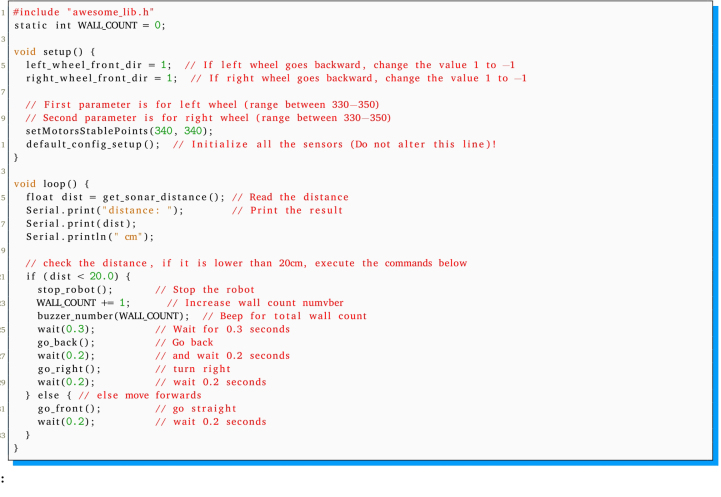

## 7 Validation and characterization

This article presents ReFiBot which is an educational robot that has been used to increase the technical literacy on robotics and bring more awareness to open science at Wageningen University. The ReFiBot combines open-source hardware and software, integrated into a chassis made from recycled plastic. The elaborate assembly instructions, design files, and software provided in this article promote the use, sharing, and modification of the ReFiBot worldwide. The low costs and various manufacturing options should also enable makers of any kind to get started with Open Source robotics.

• **Cost**: Around 35 € depending on the material used to manufacture the chassis.
• **Open Hardware**: Attached design files under GNU General Public License v3.0 on GitHub https://github.com/saidlab-team/RefiBot/tree/main/hardware.
• **Open software**: Use of Arduino and available libraries at https://github.com/saidlab-team/RefiBot/tree/main/software.
• **Manufacturability**: 3D-printable, Desktop CNC with wood or plastics. Enabling a large number to be made in a classroom setting.
• **Assembly & Instructions**: Provided in this article, with detailed renders, wiring diagrams and code examples.
• **Recyclability**: Frame can be made from recycled materials, but electronics are off-the-shelf.
• **Modularity**: Other sensors could be added with the prototyping board on top.
• **Battery life**: Lasts for 4 h during tutorial.
• **High number of sensors**: IR-line follower, RGB sensor, Ultrasonic sensor, Buzzer, Odometry sensor.

### 7.1 ReFiBot in the classroom: Student engagement and satisfaction

The educational robot has been developed for a robotics tutorial. This robotics tutorial is given to (under-) graduate students. These are students with and without a background in Computer Science and robotics at Wageningen University, as part of an Artificial Intelligence course. The aim of the tutorial is to teach the students about the working of the various peripherals on the robot and present the use cases and limitations of these peripherals. The difficulty of this tutorial is engaging the wide range of students that participate in the course. The robot does this by giving direct hands-on feedback on the code they write and the observations they need to make on the robot’s behavior. Therefore, the ReFiBot robots are ready for programming. The tutorial does not require the students to build or manufacture their own ReFiBot, rather, the tutorial covers robot programming and sensor interfacing.

This tutorial has been given twice, in January 2022 with 52 students, and in January 2023 with 44 students. The students collaborate in groups of four, following tutorial instructions, which guide the installation of the Arduino IDE and the ReFiBot library, and require the students to answer 10 questions on robot behavior, sensor principles, and guidelines for robotics. After the first year, the student response to the tutorial was overall very positive. These responses were perceived through high ratings in the course assessment, and direct contact with the students, in which said they enjoyed working with the robot and was a good change of pace within the overall structure of the course.

**Fig. 17:**
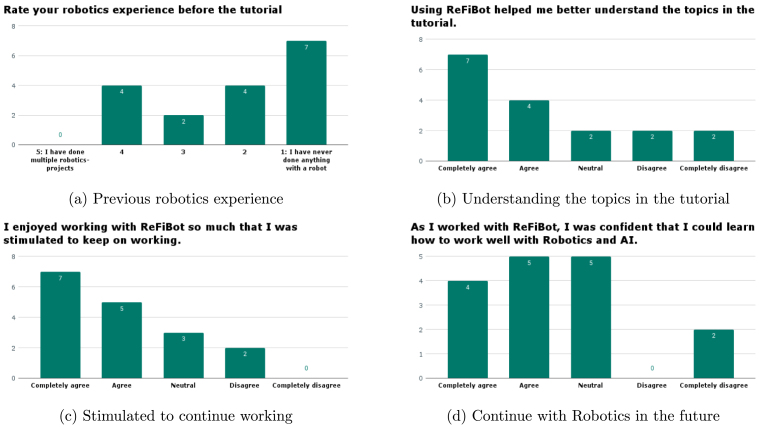
Selected responses from the questionnaire after tutorial 2.

In the second year, we requested the students to fill in a questionnaire after the tutorial about the robot and tutorial. This was to improve the tutorial for next year, and empirically gauge the response to having a robot to interact and teach with. The questionnaire was based on indicators for motivation standards by Loorbach et al. [23] and was completed by 17 students, directly after the tutorial. This results in a 19% margin of error, this uncertainty means that conclusions drawn from these numbers should be interpreted with care. However, the results are in line with similar research on motivation when including a practical robot in the classroom [2]. Most students indicated little experience with robotics (Fig. 17(a)), with some students indicating some medium experience. The response from Fig. 17(b) shows that the robot had a very positive impact on helping understand the topics in the tutorial, with a large portion on the agree-able side of the figure. For stimulation (Fig. 17(c)), most responses indicate that the robot helped the students to keep working on the tutorial. Finally, in Fig. 17(d), there was a more mixed response to the role of the educational robot in future educational activities for the students. Although the results are skewed towards the positive side. With the 17 responses, the overarching result is that the robot engaged the students as their understanding, stimulation, and future prospects around robotics were improved, even though they were not very experienced with robotics. While the questionnaire results present inconclusive findings, supplementary written feedback from the students underscores that the utilization of an actual robot for instructing robotics principles distinctly augments engagement and fosters enthusiasm towards the subject matter.

## CRediT authorship contribution statement

**Christos Pantos:** Methodology, Software, Validation, Investigation, Data curation, Writing – original draft, Writing – review & editing, Visualization. **Jurrian Doornbos:** Software, Validation, Formal analysis, Investigation, Data curation, Writing – original draft, Writing – review & editing, Visualization. **Gonzalo Mier:** Methodology, Software, Validation, Investigation, Data curation, Writing – original draft, Writing – review & editing, Visualization. **João Valente:** Conceptualization, Methodology, Validation, Resources, Writing – review & editing, Supervision, Project administration, Funding acquisition.

## Declaration of competing interest

The authors declare that they have no known competing financial interests or personal relationships that could have appeared to influence the work reported in this paper.

## Footnotes

2 For this modification the additional parts are not included in the bill of material.

## Footnotes

1 The current limitation of the USB port should be considered when designing a custom configuration with additional sensors. The battery power supply module used in this paper provides up to 2 A which is more than enough for the proposed robot configuration. Please refer to the official data sheet before proceeding with this modification.
